# A PEGylated Nanostructured Lipid Carrier for Enhanced Oral Delivery of Antibiotics

**DOI:** 10.3390/pharmaceutics14081668

**Published:** 2022-08-11

**Authors:** Seyed Ebrahim Alavi, Urooj Bakht, Maedeh Koohi Moftakhari Esfahani, Hossein Adelnia, Seyed Hossein Abdollahi, Hasan Ebrahimi Shahmabadi, Aun Raza

**Affiliations:** 1 Immunology of Infectious Diseases Research Center, Research Institute of Basic Medical Sciences, Rafsanjan University of Medical Sciences, Rafsanjan 7717933777, Iran; 2 School of Food and Agricultural Sciences, University of Management and Technology, Lahore 54770, Pakistan; 3 Australian Institute for Bioengineering and Nanotechnology, The University of Queensland, Brisbane, QLD 4072, Australia; 4 Department of Microbiology, School of Medicine, Rafsanjan University of Medical Sciences, Rafsanjan 7717933777, Iran; 5 Molecular Medicine Research Center, Rafsanjan University of Medical Sciences, Rafsanjan 7717933777, Iran; 6 School of Pharmacy, Jiangsu University, Zhenjiang 212013, China

**Keywords:** antibiotic, methicillin-resistance *Staphylococcus aureus*, nanostructured lipid carrier (NLC), PEGylation, oral delivery, trimethoprim/sulfamethoxazole

## Abstract

Antimicrobial resistance is a major concern for public health throughout the world that severely restricts available treatments. In this context, methicillin-resistant *Staphylococcus aureus* (MRSA) is responsible for a high percentage of *S. aureus* infections and mortality. To overcome this challenge, nanoparticles are appropriate tools as drug carriers to improve the therapeutic efficacy and decrease the toxicity of drugs. In this study, a polyethylene glycol (PEG)ylated nanostructured lipid carrier (PEG-NLC) was synthesized to improve the oral delivery of trimethoprim/sulfamethoxazole (TMP/SMZ) for the treatment of MRSA skin infection in vitro and in vivo. The nanoformulation (PEG-TMP/SMZ-NLC) was synthesized with size and drug encapsulation efficiencies of 187 ± 9 nm and 93.3%, respectively, which could release the drugs in a controlled manner at intestinal pH. PEG-TMP/SMZ-NLC was found efficient in decreasing the drugs’ toxicity by 2.4-fold in vitro. In addition, the intestinal permeability of TMP/SMZ was enhanced by 54%, and the antibacterial effects of the drugs were enhanced by 8-fold in vitro. The results of the stability study demonstrated that PEG-TMP/SMZ-NLC was stable for three months. In addition, the results demonstrated that PEG-TMP/SMZ-NLC after oral administration could decrease the drugs’ side-effects such as renal and hepatic toxicity by ~5-fold in MRSA skin infection in Balb/c mice, while it could improve the antibacterial effects of TMP/SMZ by 3 orders of magnitude. Overall, the results of this study suggest that the application of PEGylated NLC nanoparticles is a promising approach to improving the oral delivery of TMP/SMZ for the treatment of MRSA skin infection.

## 1. Introduction

Antimicrobial resistance (AMR) has emerged as a significant risk to global public health and severely restricts available treatments [1,2]. Statistics have demonstrated that AMR contributes to approximately 700,000 deaths worldwide annually and have estimated that this could rise to 10 million annually by 2050 [3]. Antimicrobial-resistant microorganisms are a substantial contributor to infection-related morbidity and mortality [4]. Today, antibiotics are the primary strategy for treating bacterial infections [5]. There are various bacterial mechanisms responsible for antibiotic resistance, such as (i) innate resistance, where genes are responsible for AMR; (ii) acquired resistance, where genetic mutation and/or acquisition of foreign resistance genes are responsible for AMR; (iii) adaptive resistance, where exposure to the lower doses of antimicrobials that promote genetic alterations is responsible for AMR [5].

**Figure 1:**
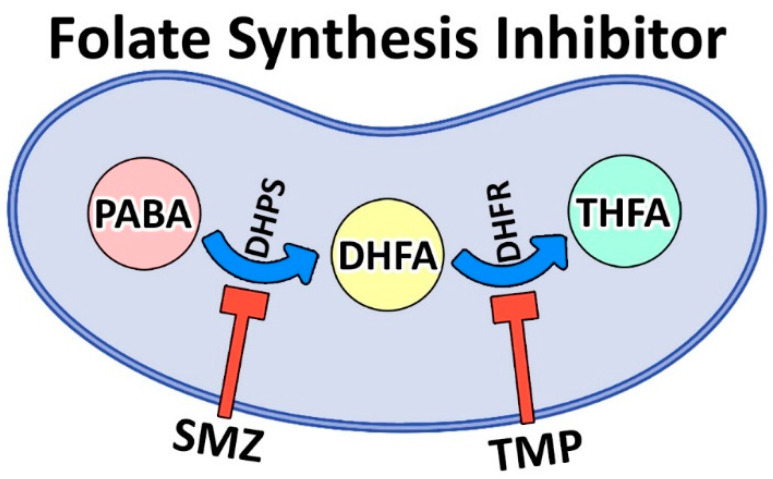
Biosynthesis pathway of folic acid from para-aminobenzoic acid (PABA) using nicotinamide adenine dinucleotide phosphate (NADPH) coenzyme. PABA, as a precursor, contributes to the synthesis of dihydrofolic acid (DHFA) and then tetrahydrofolic acid (THFA). THFA, in turn, gives rise to thymidine, purines, and methionine. TMP/SMZ inhibits folate synthesis by inhibiting dihydrofolate reductase (DHFR) and dihydropteroate synthetase (DHPS), respectively, as the two key enzymes of the folate pathway.

Methicillin-resistant *Staphylococcus (S.) aureus* (MRSA) is Gram-positive bacteria that causes either severe and difficult-to-treat infections in humans and animals that might result in death [1] or a significant financial impact on public health systems globally [5]. Trimethoprim (TMP)/sulfamethoxazole (SMZ) is considered the treatment of choice for infections brought on by MRSA. TMP/SMZ is a competitive inhibitor of dihydrofolate reductase (DHFR) and dihydropteroate synthetase (DHPS) that inhibits the synthesis of tetrahydrofolate (THF, a vital cofactor for nucleotide synthesis) and its conversion (Figure 1) [6].

However, the clinical application of TMP/SMZ is associated with various side-effects [7]. One of the promising approaches to improving the therapeutic efficacy of drugs and reducing their side-effects is the loading of drugs into nanoparticles [8,9,10,11,12]. Nanoparticles can cause a reduction in the body distribution of drugs, resulting in a reduction in the drugs’ side-effects [13]. Nanoparticles are able to increase the antibacterial effects of drugs by improving drugs’ pharmacokinetics, increasing their interaction with bacteria, and/or enhancing the drug targeting capacity [14,15]. One of the nanoparticles, as a nanotechnology-based device, is nanostructured lipid carriers (NLCs), which are composed of a mixture of solid lipids with spatially incompatible fluid/liquid with the preferred ratio of 70/30 to 99.9/0.1% [16]. These nanoparticles are synthesized to address the problems/defects of solid lipid nanoparticles (SLNs) and other colloidal carriers, such as nanoemulsions, polymeric nanoparticles, and liposomes [17]. Low loading capacity, drug leakage during storage, and high water volume are some of these defects that can be solved by the use of the unique advantages of NLCs [18]. High loading capacity, inhibition of drug leakage, improved flexibility for drug release, and adaptability with various routes of administration are some of these advantages [19,20]. In addition, NLCs are characterized by improved drug bioavailability, improved stability, easy preparation process, scale-up feasibility, biocompatibility, and improved targeting efficiency [20].

The physical and chemical properties of nanoparticles influence their pharmacokinetics and biodistribution. For instance, the size of nanoparticles and their surface charge and chemistry could increase serum protein binding, resulting in nanoparticle uptake and internalization by macrophages, which are known as the reticuloendothelial system [18]. This, in turn, results in the removal of the nanoparticles and their cargoes from the blood circulation. To enhance the blood half-life of the nanoparticles, scientists have used methods to modify or functionalize the surface of nanoparticles [21]. For this purpose, polyethylene glycol (PEG)ylation of nanoparticles is a popular method that has been widely used by researchers [22,23,24,25].

PEGylation is a process by which PEG, as a nonirritant and an inert hydrophilic polymer, is conjugated on the surface of the nanoparticles via covalent grafting, entrapping, or adsorbing [18,26]. The chains of PEG cause steric hindrance against plasma protein binding, resulting in improved stability of the nanodrug delivery systems. In addition, PEGylation improves the (i) pharmacokinetics and pharmacodynamics of nanoparticles and drugs, (ii) biodistribution and dwelling time at the action site, and (iii) therapeutic efficacy owing to increased drug concentration [18]. 

This study aimed to develop a stable nanoplatform for codelivery of TMP/SMZ via the oral route with reduced drug toxicity and improved antibacterial effects against MRSA skin infections in vitro and in vivo using PEGylated NLCs. To achieve this, TMP/SMZ-loaded PEGylated and nonPEGylated NLCs (PEG-TMP/SMZ-NLC and TMP/SMZ-NLC, respectively) were synthesized using melt emulsification and high-pressure homogenization methods. The nanoformulations were characterized in terms of size, zeta potential, morphology, and drug loading efficiency (LE%) using the Zetasizer, scanning electron microscopy (SEM), and ultra-high performance liquid chromatography (HPLC) instruments, respectively. The biological effects of the nanoparticles were then evaluated in vitro using 3-(4,5-dimethylthiazol-2-yl)-2,5-diphenyl-2H-tetrazolium bromide (MTT), in vitro human intestinal Caco-2 cells permeability, and minimum inhibitory concentration (MIC) assays. The efficacy of the formulations in decreasing the toxicity of TMP/SMZ and increasing the antibacterial effects of TMP/SMZ was evaluated in mice infected with MRSA skin infection.

## 2. Materials and Methods

### 2.1. Materials

Lecithin, monostearin, soybean oil, TMP/SMZ, phosphate-buffered saline (PBS), dialysis bag (10,000 nominal-molecular-weight cutoff (NMWCO)), acetonitrile (HPLC grade), di-potassium hydrogen phosphate, ethanol (EtOH), Hanks’ balanced salt solution (HBSS), blood agar, Mueller Hinton broth (MHB), hematoxylin and eosin (H&E), mannitol, Roswell Park Memorial Institute (RPMI)-1640 medium, penicillin and streptomycin (pen/strep) antibiotics, and fetal bovine serum (FBS) were purchased from Merck (Darmstadt, Germany). 1,2-distearoyl-sn-glycero-3-phosphoethanolamine-N-[methoxy(polyethylene glycol)-2000] (DSPE-PEG2000) was purchased from Avanti Polar Lipids (Alabaster, AL, USA). A 12-well Transwell^®^ insert (0.4 μm pore size, 1.12 cm^2^ area) was purchased from Corning (Corning, NY, USA). MRSA ATCC 33591 was obtained from the culture collection of the Iranian Research Organization for Science and Technology (IROST), Tehran, Iran. Human embryonic kidney HEK 293 cells, human colon adenocarcinoma Caco-2 cells, and male Balb/c mice (6–8 weeks old) were purchased from the Pasteur Institute of Iran (Tehran, Iran).

### 2.2. Nanostructured Lipid Carrier Preparation

PEG-TMP/SMZ-NLC and TMP/SMZ-NLC were synthesized using melt emulsification and high-pressure homogenization methods [27]. The formulation composition was optimized using different concentrations and ratios of lecithin, monostearin, soybean oil, TMP/SMZ, and DSPE-PEG2000 (Tables S1–S3). The best concentrations and ratios were selected according to the results of size, polydispersity index (PDI), and LE% (Table 1). To synthesize PEG-TMP/SMZ-NLC, lecithin as the solid lipid, monostearin and soybean oil as the liquid lipid, TMP/SMZ (1:5 weight ratio), and DSPE-PEG2000 were mixed and stirred (1 h, 75 °C). The aqueous phase (Tween 80 (0.1% *w*/*v*), Poloxamer 188 (0.2% *w*/*v*), and water (95.2% *w*/*v*)) was added dropwise to the lipid phase under stirring (200 RPM), and a coarse emulsion was obtained after 1 h. The emulsion was converted to a fine emulsion by mixing at 15,000 RPM using an ULTRA-TURRAX T-25 instrument (60 °C) and then homogenized (60 °C, 800 bars, 10 min) using a Lab-60 high-pressure homogenizer (APV Gaulin, Lübeck, Germany). The non-PEGylated NLCs (TMP/SMZ-NLC) were prepared with the same method, except that DSPE-PEG2000 was not added to the reaction medium.

### 2.3. Nanoparticles Characterization

#### 2.3.1. Dynamic Light Scattering

The size, size distribution, and zeta potential of the nanoparticles were measured using the Zetasizer instrument. For this purpose, a suspension of the nanoparticles (100 μg/mL) was prepared in PBS (pH 7.4, 25 °C) and then introduced into the instrument (ZEN 3600, Malvern Instruments Ltd., Worcestershire, UK).

#### 2.3.2. Scanning Electron Microscopy

The nanoparticles were morphologically evaluated under vacuum conditions using an SEM microscope. For this purpose, 200 µL of the suspensions of nanoparticles were centrifuged (13,000 RPM, 30 min, 4 °C), and the pellets were resuspended in 200 µL of mannitol solution (50 mg/mL), as a cryoprotectant, and freeze-dried using Labconco-Freezone 25. The powder of the nanoparticles was then coated with a thin layer of gold and the nanoparticles were evaluated by the SEM instrument (XL30, Philips, AE Eindhoven, The Netherlands).

#### 2.3.3. Drug Encapsulation and Loading Efficiencies

Drug encapsulation (EE%) and LE% efficiencies were measured using ultra-high performance liquid chromatography (UHPLC; Agilent Technologies Inc. Santa Clara, CA, USA) [28]. Briefly, the suspensions of nanoparticles were centrifuged (13,000 RPM, 30 min, 4 °C) and the supernatants were obtained. The drug concentration in the supernatants was obtained using UHPLC equipped with the analytical C-18 reversed-phase column (Nucleodur 18, 25 cm, 4.6 mm, 5 μm) and photon diode array (PDA) detector. The elution was performed with a mobile phase of di-potassium hydrogen phosphate (10 mM, pH 7.2) containing acetonitrile (80:20) with a flow rate of 1 mL/min. Chromatograms were reanalyzed by Agilent ChemStation software. TMP and SMZ were detected at 270 and 254 nm, respectively. After the determination of the drug concentration in the supernatant, EE% and LE% were calculated using the formulae below:

(1) $$\mathrm{EE}\%=\frac{\mathrm{Initial}\;\mathrm{drug}\;\mathrm{concentration}\;\left(\mathrm{mg}\right)-\mathrm{Drug}\;\mathrm{concentration}\;\mathrm{in}\;\mathrm{supernatant}\;\left(\mathrm{mg}\right)}{\mathrm{Initial}\;\mathrm{drug}\;\mathrm{concentration}\;\left(\mathrm{mg}\right)}\times 100\;$$

(2) $$\mathrm{LE}\%=\frac{\mathrm{Loaded}\;\mathrm{drug}\;\mathrm{in}\;\mathrm{nanoparticles}\;\left(\mathrm{mg}\right)}{\mathrm{Weight}\;\mathrm{of}\;\mathrm{nanoparticles}\;\left(\mathrm{mg}\right)}\times 100\;$$

### 2.4. Release Study

To determine the effects of PEGylation on the profile of drug release from the nanoparticles and determine the efficacy of the nanoparticles for the oral drug delivery, the drug release study was performed using the dialysis membrane method at pH 1.2 and 6.8 to simulate the pH of gastric and intestinal environments, respectively [29]. For this purpose, the suspensions of TMP/SMZ-NLC and PEG-TMP/SMZ-NLC were centrifuged to obtain the precipitates of the nanoparticles. The precipitates equal to 3 mg of TMP/SMZ were resuspended into 5 mL of PBS (pH 1.2 and 6.8) and transferred into 4 separate dialysis bags. The bags were then immersed in 100 mL of PBS (pH 1.2 and 6.8), as the acceptor medium, and stirred (150 RPM, 25 °C). Two separate solutions of TMP/SMZ at the concentration of 3 mg/5 mL PBS were prepared, transferred to two separate dialysis bags, immersed into 100 mL of PBS (pH 1.2 and 6.8), as the acceptor medium, and stirred (150 RPM, 25 °C). At the predetermined time intervals (0.5, 1, 2, 4, and 8 h), 1 mL of the buffer was withdrawn and replaced with the fresh buffer. The drug concentrations in the collected samples were measured using UHPLC, and the cumulative drug release versus time was measured using the formula below:

(3) $$\mathrm{Drug}\;\mathrm{release}\;\left(\%\right)=\frac{\mathrm{Mass}\;\mathrm{of}\;\mathrm{the}\;\mathrm{released}\;\mathrm{drug}\;\mathrm{from}\;\mathrm{nanoparticles}\;\left(\mathrm{mg}\right)}{\mathrm{Mass}\;\mathrm{of}\;\mathrm{the}\;\mathrm{loaded}\;\mathrm{drug}\;\mathrm{in}\;\mathrm{nanoparticles}\;\left(\mathrm{mg}\right)}\times 100\;$$

The kinetics of drug release were also determined by the use of zero-order, first-order, Higuchi, and Korsmeyer–Peppas mathematical models [30].

### 2.5. Evaluation of the Biological Effects of the Nanoparticles

#### 2.5.1. Evaluation of the Toxicity of the Nanoparticles 

The toxicity of PEG-TMP/SMZ-NLC and TMP/SMZ-NLC, compared to the standard TMP/SMZ, was evaluated using human embryonic kidney HEK293 cells and MTT assay. For this purpose, the cells were seeded at the density of 10^4^ cells/well in 96-well plates containing 100 µL of RPMI-1640 culture medium supplemented with 10% FBS and 1% pen/strep antibiotics (complete media). The plates were then transferred to a 5% CO_2_ incubator and incubated for 24 h at 37 °C. The culture media was removed; 100 µL of the fresh complete culture media, containing 2, 4, 8, 16, 32, 64, 128, and 256 µM of TMP/SMZ in the standard form and encapsulated into the nanoparticles (TMP/SMZ-NLC and PEG-TMP/SMZ-NLC), were added to the wells; the cells were incubated for 48 h in the incubator (5% CO_2_ and 37 °C). The culture media was then replaced with 100 µL of MTT solution (0.5 mg/mL in PBS), and the cells were incubated for 3 h at 37 °C. The MTT solution was discarded, and 100 µL of DMSO was added to each well to dissolve the formazan crystals and was incubated for 20 min. The absorbance was read at 570 nm using a microplate reader, and the cell viability was calculated using the following formula:

(4) $$\mathrm{Cell}\;\mathrm{viability}\;\left(\%\right)=\frac{{\mathrm{Absorbance}}_{\mathrm{sample}}-{\mathrm{Absorbance}}_{\mathrm{background}}}{{\mathrm{Absorbance}}_{\mathrm{negative}\;\mathrm{control}\;}-{\;\mathrm{Absorbance}}_{\mathrm{background}}}\times 100$$

The negative and positive controls were the cells incubated with the complete media, and the cells were treated with SDS (10% *v*/*v* in water) + 0.1 M HCl, respectively. In addition, the background was the complete media only. The half-maximal inhibitory concentration (IC_50_) of TMP/SMZ, TMP/SMZ-NLC, and PEG-TMP/SMZ-NLC was calculated using GraphPad Prism Software version 8.00 (GraphPad Software, Inc., San Diego, CA, USA).

#### 2.5.2. Stability Study

The stability of PEG-TMP/SMZ-NLC, compared to TMP/SMZ-NLC, was measured using the dialysis bag, MTT assay, DLS, and UHPLC methods, 3 months after their preparation. Briefly, 5 mL of PEG-TMP/SMZ-NLC and TMP/SMZ-NLC equivalent to 238.5 and 206.5 mg of the nanoparticles, respectively, were stored in a 4 °C refrigerator for 3 months, and their profile of drug release, in vitro toxicity, size, PDI, and LE% was measured using the methods mentioned above. In addition, the serum stability of PEG-TMP/SMZ-NLC, compared to TMP/SMZ-NLC, was measured in FBS [31]. Briefly, a suspension of 10 mg/mL from TMP/SMZ-NLC and PEG-TMP/SMZ-NLC was prepared in FBS:PBS (45:55% *v*/*v* ratio) and incubated for 5 h at 37 °C Next, the size of the formulations was calculated at the time intervals of 1, 3, and 5 h.

#### 2.5.3. In Vitro Caco-2 Permeability

The permeability rate of TMP/SMZ-NLC and PEG-TMP/SMZ-NLC, compared to TMP/SMZ, was measured using Caco-2 cells. The cells were cultured in the complete media and incubated (5% CO_2_ and 37 °C) to reach 90% confluency. The cells were then trypsinized and suspended at the concentration of 2 × 10^5^/mL in the complete media. The cells (0.5 mL: 1 × 10^5^/mL) were cultured on the apical chamber (A) of a 12-well transwell insert, and 1.5 mL of the fresh medium was added into the basolateral chamber (B) of each insert.

##### Determining the Transepithelial Electrical Resistance

The electrode of the volt-ohmmeter was incubated with the complete culture medium for 20 min to equilibrate. In addition, the culture medium of the apical and basolateral chambers of the insert plates was replaced with the fresh complete culture medium every 48 h. After 20 min incubation at 37 °C, the transepithelial electrical resistance (TEER) was recorded. The cell monolayer with TEER ≤ 500 cm^2^ Ω was considered an appropriate cell monolayer for subsequent experiments. The amounts of TEER of the Caco-2 cell monolayer were measured using the formula below:

$${\mathrm{TEER}\;(\mathrm{cm}}^{2}\;Ω)=\left[\mathrm{TEER}\;(Ω)\;-\;\mathrm{TEERbackground}\;(Ω)\right]\times {\;A\;(\mathrm{cm}}^{2})$$

where TEER (Ω), TEERbackground (Ω), and A are the electrical resistance of Caco-2 cell monolayers, the resistance of the insert without the cells, and the surface area of the insert (1.12 cm^2^), respectively. 

##### Evaluation of the Nanoparticles’ Permeability

In this experiment, Caco-2 monolayer cells with the TEER ≤ 500 cm^2^ Ω were formed within 6 days and used for the permeability study. To evaluate the permeability, the medium of each well was discarded, and preheated HBSS (washing medium of cell monolayers) was added to each well and incubated for 20 min at 37 °C. Next, the media was discarded, and 0.5 mL of HBSS containing 125 µg of TMP/SMZ in the standard form and encapsulated into the nanoparticles (TMP/SMZ-NLC (1202 µg) and PEG-TMP/SMZ-NLC (1276 µg)) was added in chamber A, while 1.5 mL of fresh HBSS was added to chamber B. At the time intervals of 1.5, 3, 4.5, and 6 h, 0.2 mL of the medium in chamber B was withdrawn and replaced with the fresh HBSS. The drug concentration in the collected samples was measured and compared using UHPLC.

#### 2.5.4. In Vitro Bacterial Assay

To determine the MIC of PEG-TMP/SMZ-NLC, compared to TMP/SMZ-NLC and TMP/SMZ, a broth microdilution method and an MRSA bacterium (ATCC 33591) were used. For this purpose, TMP/SMZ, TMP/SMZ-NLC, and PEG-TMP/SMZ-NLC were serially twofold diluted with MHB to obtain the drug concentrations of 2, 4, 8, 16, 32, 64, 128, and 256 µM of TMP/SMZ. An amount of 100 µL of the dilutions was plated into flat-bottom microtiter plates. A 0.5 McFarland bacterial suspension (1 × 10^8^ colony-forming unit (CFU)/mL) was prepared in sterile-distilled water and diluted (1:100) into MHB to obtain 1 × 10^6^ CFU/mL inoculum. An amount of 100 µL of the inoculum was then added to the TMP/SMZ samples to prepare a final initial inoculum of 0.5 × 10^6^ CFU/mL and incubated for 20 h at 37 °C. The bacterial suspension without antibiotics prepared in MHB and the MHB culture medium only were considered as the negative and sterile control, respectively. The MIC values were determined spectrophotometrically at 620 nm. The experiment was repeated three times.

#### 2.5.5. Animal Study

To evaluate the therapeutic efficacy of PEG-TMP/SMZ-NLC, compared to TMP/SMZ-NLC and TMP/SMZ, for the treatment of MRSA skin infection, male Balb/c mice (6–8 weeks) were used. The experiments were approved by the Ethic Committee of Rafsanjan University of Medical Sciences, Rafsanjan, Iran (approval code: IR.RUMS.REC.1399.89; approval date: 7 August 2020). The animals were maintained in standard and controlled conditions of temperature (25 ± 2 °C), humidity (50–60%), and light (12 h light/dark cycle). They were maintained in polypropylene cages and allowed free access to food and water. The bottom of the cages was lined with wood husks, which were changed frequently. After 1 week of acclimation, the hair on the back was shaved, and the skin was disinfected with 70% EtOH. The mice were then anesthetized intraperitoneally with a mixture of xylazine (8 mg/kg) and ketamine (50 mg/kg). A round wound (5 mm diameter) was created on the back of each mouse using a biopsy punch. After 5 min, the wound was covered with a small piece of gauze and inoculated with 50 µL of bacterial suspension containing 10^8^ CFU prepared in PBS. The wound was closed with skin clips, and the mice were returned to the cages. At 48 h after wound creation, the animals were randomly divided into 4 groups (n = 10) and received TMP/SMZ, TMP/SMZ-NLC, PEG-TMP/SMZ-NLC, and PBS, as the control group. The formulations were administered orally at the drug dose of 20 mg/kg every 24 h and for 10 days. After 10 days, the mice were sacrificed, and the skin containing the wound was removed and inspected in terms of the number of viable bacteria. Briefly, the tissues were homogenized in 1 mL of sterile PBS and diluted with distilled water. Next, the solutions were serially diluted and cultured in blood agar plates. The plates were maintained for 48 h at 37 °C, and the number of viable bacteria was calculated by CFU counting. In addition, the side-effects of the formulations were evaluated by measuring the weight changes of the animals, hepatic (alanine aminotransferase (ALT) and aspartate aminotransferase (AST)) and renal serum markers (blood urea nitrogen (BUN) and creatinine), and histopathological studies.

### 2.6. Statistical Analysis

All statistical analyses were performed using GraphPad Prism software version 8.00, San Diego, CA, USA. The data of size, PDI, LE%, EE%, release study, toxicity of the nanoparticles, in vitro permeability, and in vitro bacterial assay were expressed as the mean ± standard deviation (SD, n = 3). The one-way analysis of variance (ANOVA) test was used to analyze all statistical differences. In addition, statistical analysis was performed using nonlinear regression analysis.

## 3. Results and Discussion

### 3.1. Nanoparticle Characterization

NLC, PEG-NLC, TMP/SMZ-NLC, and PEG-TMP/SMZ-NLC were successfully synthesized using melt emulsification and high-pressure homogenization methods. The size, PDI, and zeta potential of the nanoparticles were measured using the Zetasizer instrument. The results showed that all of the formulations were synthesized at the nanoscale size (Table 2), in which the size of NLC, PEG-NLC, TMP/SMZ-NLC, and PEG-TMP/SMZ-NLC was 184 ± 7, 170 ± 10, 198 ± 11, and 187 ± 9 nm, respectively. All the formulations demonstrated a negative zeta potential (−25 ± 1.4, −20 ± 1.3, −19 ± 1, and −13 ± 0.8 mV for NLC, PEG-NLC, TMP/SMZ-NLC, and PEG-TMP/SMZ-NLC, respectively), causing them to repel each other, and consequently, inhibiting aggregation. In addition, the formulations demonstrated PDI values in the range of 0.1–0.4, confirming that they were monodisperse and homogeneous [30,32]. The particles were PEGylated to improve the blood half-life and biocompatibility [33,34], solubility, tumor-targeting efficiency [26,35,36,37], drug release profile [38], stability [39,40], and oral absorption [41]. PEG works as a lamellarity reducing agent and improves stability, which causes an inhibition in the nanoparticle aggregation [42,43,44].

PEGylated nanoparticles (PEG-NLC and PEG-TMP/SMZ-NLC), compared to the non-PEGylated counterparts (NLC and TMP/SMZ-NLC), were smaller and demonstrated lower PDI values (Table 2). Lakhani et al. [27] also demonstrated that PEGylated NLCs, compared to non-PEGylated NLCs, were smaller (218 vs. ~570 nm). This could be due to PEG’s capability to decrease the lamellarity and increase the stability of the conjugate [43,44]. PEGylated nanoparticles, compared to the non-PEGylated nanoparticles, also demonstrated an increase in the zeta potential that could result from the neutral charge of PEG. The difference in the Zeta potential between PEGylated and non-PEGylated nanoparticles indicated that the nanoparticles were successfully PEGylated.

**Figure 2:**
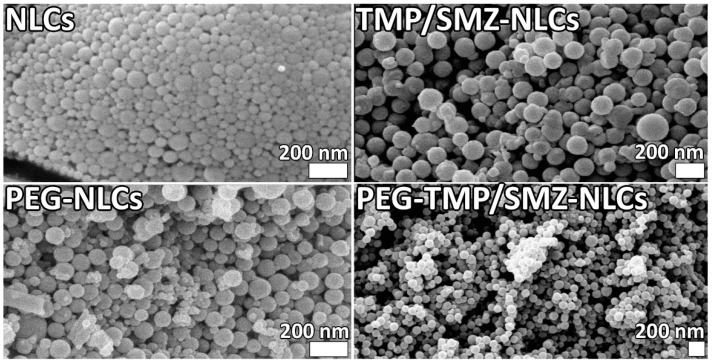
Scanning electron microscopy (SEM) of NLCs, TMP/SMZ-NLCs, PEG-NLCs, and PEG-TMP/SMZ-NLCs.

In addition, the morphology of NLCs, TMP/SMZ-NLCs, PEG-NLCs, and PEG-TMP/SMZ-NLCs was evaluated and compared using an SEM instrument. The results of SEM confirmed the synthesis of the nanoparticles as spherical particles with smooth surfaces (Figure 2). In addition, the results of SEM demonstrated that the nanoparticles were synthesized in a homogenous and monodisperse mode.

In addition, the EE% and LE% of TMP/SMZ-NLCs and PEG-TMP/SMZ-NLCs were measured using UHPLC at 270 and 254 nm. The EE% and LE% for TMP/SMZ-NLCs and PEG-TMP/SMZ-NLCs were found to be 86.2 and 10.4, and 93.3% and 9.8%, respectively (Table 2). As the results showed, PEGylated nanoparticles (PEG-TMP/SMZ-NLCs), compared to the non-PEGylated ones (TMP/SMZ-NLCs), caused a higher EE% (93.3 vs. 86.2%). This could be due to the function of PEG, as PEG could cause a change in the hydrophobicity, rigidity, chain order, and spacing between tails of the lipid membrane and, as a result, cause an increase in the EE% [45]. These results were in agreement with the results of the Zhang et al. study, [46] in which PEGylated NLCs, compared to nonPEGylated NLCs, caused a higher EE% (92.2 vs. 88.6%).

### 3.2. Release Study

**Figure 3:**
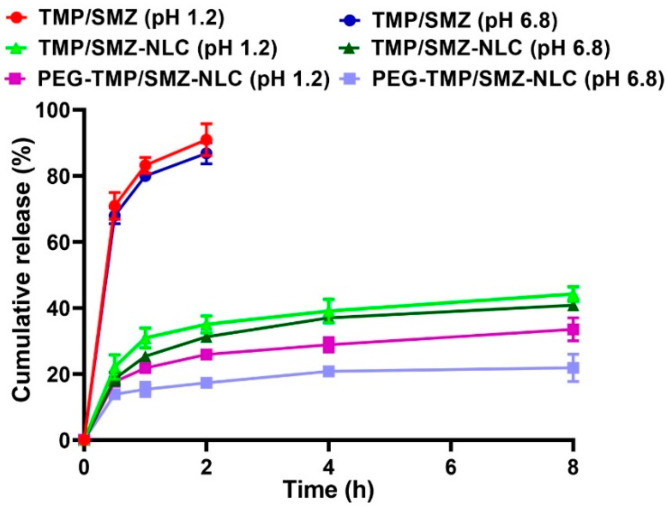
Cumulative drug release from TMP/SMZ solution, TMP/SMZ-NLCs, and PEG-TMP/SMZ-NLCs. As the figure shows, PEG-TMP/SMZ-NLCs, compared to TMP/SMZ-NLCs, caused a significant decrease in the amount of drug release (*p* < 0.05). Results are expressed as mean ± SD of three independent experiments.

The efficacy of PEG-TMP/SMZ-NLCs, compared to TMP/SMZ-NLCs, in controlling the drug release was evaluated using a dialysis bag method. The drug release from the nanoparticles was evaluated at two pHs, 1.2 and 6.8, to simulate the gastric and intestinal environments, respectively [47]. According to the results, the drug release at both pHs demonstrated two distinct phases, including an initial burst release, followed by a slow and sustained release. In the burst phase, 22 and 18% of the loaded drug were released at pH 1.2 and 6.8 from TMP/SMZ-NLCs in the first 30 min of the study, while these values for PEG-TMP/SMZ-NLCs were 17 and 13%, respectively. These burst releases could be due to the release of the adsorbed drug [35] that continued with a slow and sustained release, in which 43.2 and 40% of the loaded drug was released from TMP/SMZ-NLCs at pH 1.2 and 6.8, respectively, after 8 h, while these values for PEG-TMP/SMZ-NLCs were 32.4 and 26.7%, respectively (Figure 3). According to these results, PEG-TMP/SMZ-NLCs, compared to TMP/SMZ-NLCs, were more efficient by 25 and ~33.2% at pH 1.2 and 6.8, respectively, in preserving the loaded drug and released them for a longer time. Coating the nanoparticles’ surface with PEG restrains the leakage of the loaded drug from the particles. Premature drug release limits the nanoparticles’ application [48] that could be resolved through PEGylation. In agreement with the results of the present study, Chime et al. [49] demonstrated that PEGylated NLCs, compared to non-PEGylated NLCs, caused a lower amount of drug release (~33.5 vs. ~48%). In addition, the results of drug release from TMP/SMZ solutions demonstrated that 91 and 100% of the standard drug were released from the solutions after 2 and 2.5 h, respectively, at pH 1.2, while these values for pH 6.8 were 87 and 100%, respectively (Figure 3), indicating the high efficacy of TMP/SMZ-NLCs and PEG-TMP/SMZ-NLCs to control the release of the drug [48]. Overall, the results of the drug release study demonstrated that NLCs and PEGylated NLCs provided controlled drug release systems. In addition, PEGylation of nanoparticles caused a further improvement in the profile of drug release at both gastric and intestinal pHs; thus, PEG-TMP/SMZ-NLCs could be considered for oral delivery of TMP/SMZ to improve its therapeutic effects.

The kinetics of drug release from TMP/SMZ-NLCs and PEG-TMP/SMZ-NLCs were also measured using zero-order, first-order, Higuchi, and Korsmeyer–Peppas mathematical models. In the kinetic model of zero-order, the drug release rate from the polymer matrix is constant throughout an experiment and is not dependent on the drug concentration; therefore, the same amount of drugs per unit of time is released over time. However, in the kinetic model of first-order, the drug release rate is dependent on the drug concentration and, thus, decreases with time [50]. In the kinetic model of Higuchi, the amount of cumulative released drug is directly proportional to the square root of time [51], and in the Korsmeyer–Peppas model, the rate of drug release is tuned by the diffusion and swelling rate [52,53]. The results of the present study demonstrated that TMP/SMZ-NLCs and PEG-TMP/SMZ-NLCs fitted well with the Higuchi model with the correlation coefficient (R^2^) values of 0.8269 and 0.8835, respectively, at pH 1.2, and 0.8653 and 0.8789, respectively, at pH 6.8 (Figure S1, Tables S4–S7).

### 3.3. Evaluation of the Biological Effects of the Nanoparticles

#### 3.3.1. Evaluation of the Toxicity of the Nanoparticles

**Figure 4:**
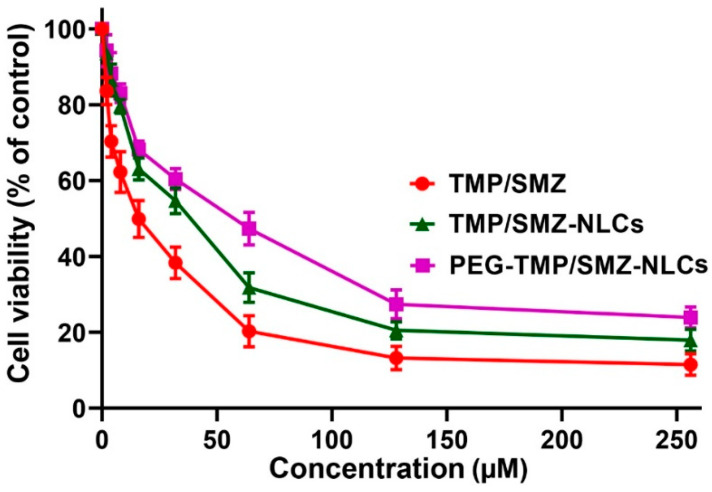
Cell toxicity effects of TMP/SMZ-NLCs and PEG-TMP/SMZ-NLCs, compared to TMP/SMZ, against human embryonic kidney HEK293 cells, measured by MTT assay. The results were expressed as mean ± SD (n = 3).

The toxicity effects of PEG-TMP/SMZ-NLCs, compared to TMP/SMZ-NLCs and TMP/SMZ, were measured in vitro using an MTT assay and HEK293 cells. In addition, the toxicity effects of NLCs and PEG-NLCs at different concentrations (e.g., 5, 10, 20, 40, and 80 mg/mL) were evaluated to determine their nontoxic concentrations. The results demonstrated that NLCs and PEG-NLCs at the concentration of 10 mg/mL were safe and nontoxic. In addition, the results demonstrated that TMP/SMZ-NLCs and PEG-TMP/SMZ-NLCs could reduce the TMP/SMZ toxicity by 1.5- and 2.4-fold, respectively (IC_50_: 25.4, 40.9, and 16.9 µM for TMP/SMZ-NLCs, PEG-TMP/SMZ-NLCs, and TMP/SMZ, respectively; Figure 4). The higher efficacy of PEG-TMP/SMZ-NLCs, compared to TMP/SMZ-NLCs, in reducing the drugs’ toxicity could result from the profile of drug release from PEG-TMP/SMZ-NLCs, in which PEG-TMP/SMZ-NLCs, compared to TMP/SMZ-NLCs, were more efficient in preserving the loaded drugs and their release for a longer time.

#### 3.3.2. Stability Study

**Figure 5:**
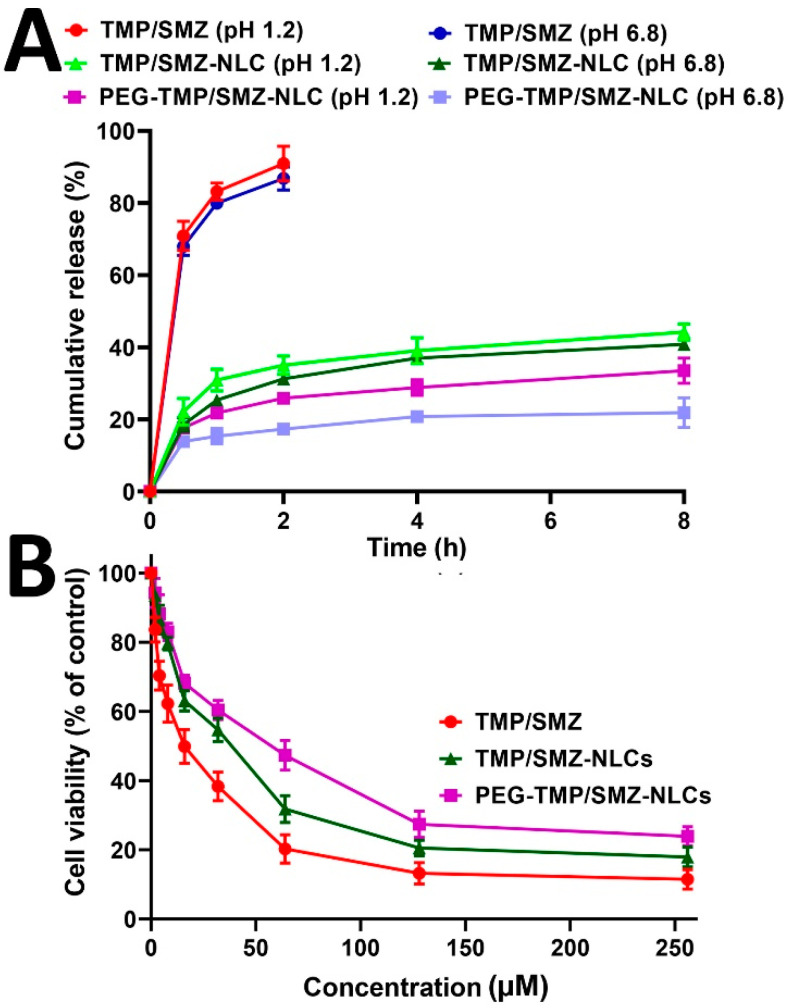
(**A**) Cumulative drug release from TMP/SMZ-NLCs and PEG-TMP/SMZ-NLCs, and (**B**) cell toxicity effects of TMP/SMZ-NLCs and PEG-TMP/SMZ-NLCs, compared to TMP/SMZ, 3 months after the preparation. The data were expressed as mean ± SD (n = 3).

The stability of nanoparticles is a critical factor in their synthesis, storage, and efficacy for the therapeutics delivery to blood circulation [54,55]. The sufficient stability of nanoparticles is required to preserve their cargos under harsh biological environments, such as low pH and enzymatic decomposition [56]. For this reason, the profile of drug release, in vitro toxicity, and drug loading efficiency of TMP/SMZ-NLCs and PEG-TMP/SMZ-NLCs were measured and compared with those obtained in the production time. According to the obtained results, TMP/SMZ-NLCs and PEG-TMP/SMZ-NLCs preserved their efficacy in controlling the drug release, in which TMP/SMZ-NLCs and PEG-TMP/SMZ-NLCs released 91.2% and 67.2% of the loaded drug, respectively, at pH 1.2 after 52 h and 76.3% and 54.5% of the loaded drug, respectively, at pH 6.8 after 52 h. These values were 86% and 64%, respectively, at pH 1.2 and 73% and 52%, respectively, at pH 6.8 in the production time (Table 3, Figure 5A). In addition, the values of LE% measured three months after the production time did not significantly change compared to those obtained at the time of production, in which LE% for TMP/SMZ-NLCs and PEG-TMP/SMZ-NLCs was 9.7% and 9.1%, respectively, while these values at the time of preparation were 10.4% and 9.8%, respectively (Table 3). Moreover, the cell toxicity results did not significantly change compared to those obtained in the production time, in which the IC_50_ values for TMP/SMZ-NLCs and PEG-TMP/SMZ-NLCs were 27.8 and 43.1 µM, respectively, while these values at the production time were 25.4 and 40.9 µM, respectively (Figure 5B). 

In addition, the stability of TMP/SMZ-NLCs and PEG-TMP/SMZ-NLCs was measured in terms of the size and PDI of the formulations, and the results demonstrated that the size and PDI of TMP/SMZ-NLCs and PEG-TMP/SMZ-NLCs, 3 months after the production, were comparable to those results obtained at the production time (Table 4). However, PEG-TMP/SMZ-NLCs, compared to TMP/SMZ-NLCs, were found to be more potent in preserving their size (~4% vs. ~7% size increment for PEG-TMP/SMZ-NLCs and TMP/SMZ-NLCs, respectively). In addition, the stability of TMP/SMZ-NLCs and PEG-TMP/SMZ-NLCs was evaluated in an FBS solution, and the results demonstrated that PEG-TMP/SMZ-NLCs, compared to TMP/SMZ-NLCs, were more stable in this serum solution (size increment of 12.5 and 20.3% for PEG-TMP/SMZ-NLCs and TMP/SMZ-NLCs, respectively; Figure S2). This result was in agreement with the results of size and PDI measurements in that PEG-TMP/SMZ-NLCs were found to be more stable compared to TMP/SMZ-NLCs.

According to these results, TMP/SMZ-NLCs and PEG-TMP/SMZ-NLCs were stable carriers for TMP/SMZ and, thus, could improve the stability of TMP/SMZ. These results are in agreement with the results of Lakhani et al.’s study [27], where they demonstrated that amphotericin B(AmB)-loaded PEGylated NLCs, compared to AmB-loaded non-PEGylated NLCs, were more stable, in which lower increments (~1% vs. ~55%) in the size values were observed for AmB-loaded PEGylated NLCs, compared to that of AmB-loaded non-PEGylated NLCs, after autoclaving of the nanoparticles.

#### 3.3.3. Evaluation of the Nanoparticles’ Permeability

**Figure 6:**
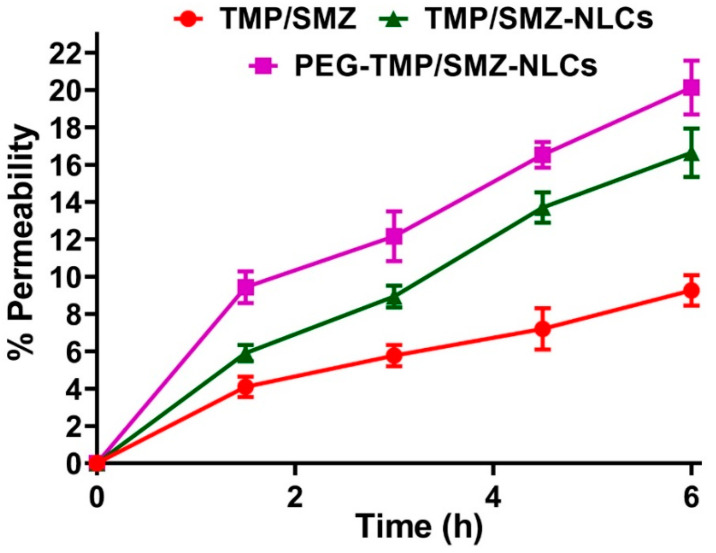
The TMP/SMZ transported through the Caco-2 cell monolayer quantified using UHPLC. The results were expressed as mean ± SD from 3 independent experiments (n = 3).

To determine the permeability of PEG-TMP/SMZ-NLC, TMP/SMZ-NLC, and TMP/SMZ across the gastrointestinal tract, a Caco-2 monolayer culture model was used as the Caco-2 monolayer culture model is well-established as having absorptive (A to B) characteristics of intestinal epithelial cells [57]. Thus, the directional transport of TMP/SMZ in the standard form and encapsulated into NLCs and PEG-NLCs was measured across the Caco-2 monolayer at four different time points (1.5, 3, 4.5, and 6 h). As the results demonstrated (Figure 6), the encapsulation of TMP/SMZ into NLCs and PEG-NLCs caused an increase in the transport of TMP/SMZ across the cell monolayer; however, PEG-TMP/SMZ-NLC, compared to TMP/SMZ-NLC and TMP/SMZ, demonstrated a higher efficacy to transport across the cell monolayer by 17.4 and 54%, respectively. This could be due to the smaller size of the PEGylated nanoparticles, compared to nonPEGylated ones, as transport is a size-dependent process [58]. In addition, as PEGylated nanoparticles are more stable and less likely to aggregate, they are more in favor of transcytosis [59]. The high efficacy of PEGylated nanoparticles to increase the intestinal permeability of a drug has also been demonstrated previously [60], where the encapsulation of paclitaxel into pegylated poly(anhydride) nanoparticles caused a 3–7-times increase in the intestinal permeability of the drug.

#### 3.3.4. In Vitro Bacterial Assay

Nanoparticles, as drug carriers, can improve drugs’ therapeutic effects [30,35]. To determine the efficacy of PEG-TMP/SMZ-NLCs, compared to TMP/SMZ-NLCs, to increase the antibacterial effects of TMP/SMZ against an MRSA bacterium, a broth microdilution method was used. The results demonstrated that PEG-TMP/SMZ-NLCs and TMP/SMZ-NLCs caused an 8- and a 4-fold decrease in the MIC value, respectively (MIC: 4, 8, and 32 µM for PEG-TMP/SMZ-NLCs, TMP/SMZ-NLCs, and TMP/SMZ, respectively), indicating the efficacy of the nanoparticles to increase the antibacterial effects of TMP/SMZ. The enhanced antibacterial effects of PEG-TMP/SMZ-NLCs, compared to TMP/SMZ-NLCs, could result from the positive surface charge of PEG-TMP/SMZ-NLCs, compared to TMP/SMZ-NLCs, that lead to PEG-TMP/SMZ-NLCs interacting more efficiently with the MRSA bacterium with the negative-charged cell membrane [61], and consequently, stronger antibacterial effects. Furthermore, the PEGylation of nanoparticles can improve the stability of the particles that, in turn, can improve the particles’ interaction with the bacterial cells [62]. In addition, PEGylation could cause an improvement in the bacterial cell permeability, leading to an increase in the nanoparticles concentration inside the cells and, as a result, the antibacterial effects of the loaded-antibacterial agents [63]. In addition, the higher drug encapsulation efficiency of PEG-TMP/SMZ-NLCs (86.2 vs. 93.3%) and slower drug release from PEG-TMP/SMZ-NLCs (18 vs. 13%), compared to TMP/SMZ-NLCs, could be the reasons for increasing the antibacterial effects of PEG-TMP/SMZ-NLCs [63].

#### 3.3.5. Animal Study

**Figure 7:**
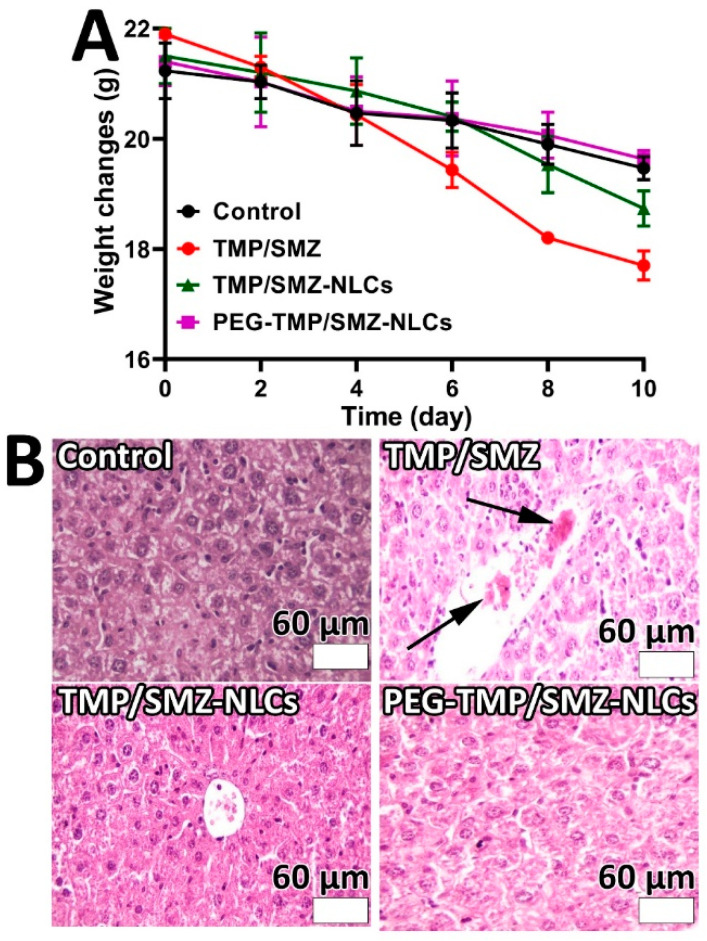
(**A**) Weight change measurements in MRSA skin infections in Balb/c mice 10 days after the development of infection. As the figure shows, TMP/SMZ receiver mice, compared to TMP/SMZ-NLCs, PEG-TMP/SMZ-NLCs receiver, and the control groups of mice, demonstrated higher weight loss. (**B**) Histopathological effects of (i) TMP/SMZ, (ii) TMP/SMZ-NLCs, and (iii) PEG-TMP/SMZ-NLCs, compared to (iv) PBS, on the liver of the skin-infected mice caused by MRSA. Arrows show the histopathological lesion (Magnification ×40).

MRSA is the cause of the highest proportion of *S. aureus* infections (up to 55%) and mortality (20%) [64]. Thus, it is recommended to develop more effective therapeutic strategies to improve clinical outcomes [65]. For this purpose, the application of nanotechnology-based devices can be considered a promising strategy to address these issues. In this study, the therapeutic efficacy of PEG-TMP/SMZ-NLCs, compared to TMP/SMZ-NLCs and TMP/SMZ, in treating MRSA skin infections was measured and compared in vivo. For this purpose, the weight changes of the infected animals were controlled and recorded throughout the experiment. According to the results, the weight loss occurring in the TMP/SMZ receiver group was more prominent compared to the other groups (8, 8.4, 13, and 19.2% weight loss in control, PEG-TMP/SMZ-NLCs, TMP/SMZ-NLCs, and TMP/SMZ receiver groups, respectively, Figure 7). This results from the efficacy of nanoparticles in decreasing the side-effects of drugs as the nanoparticles are able to reduce the drug distribution in the body [13].

**Figure 8:**
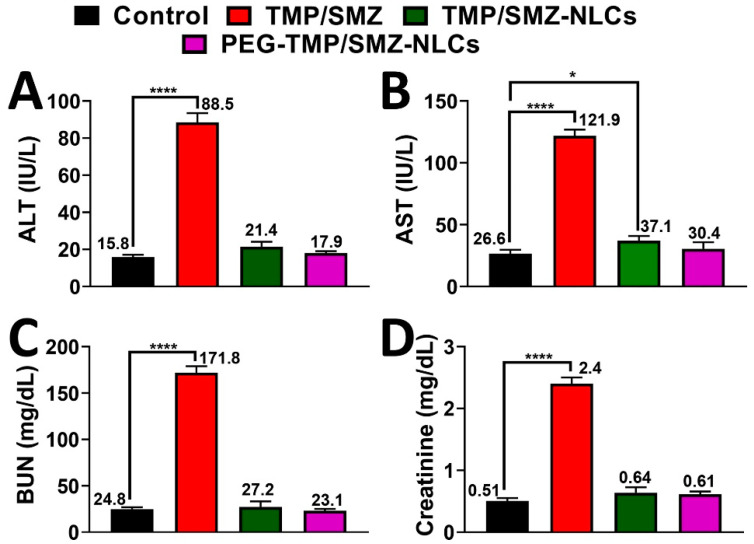
Serum levels of (**A**) alanine aminotransferase (ALT), (**B**) aspartate aminotransferase (AST), (**C**) BUN, and (**D**) creatinine in TMP/SMZ, TMP/SMZ-NLCs, and PEG-TMP/SMZ-NLCs groups compared to the control group. The results were expressed as mean ± SD from three independent experiments. * and **** indicate *p* < 0.05 and <0.0001, respectively, compared with the control group.

In addition, the serum concentrations of ALT, AST, BUN, and creatinine were measured in the infected animals, and the results demonstrated that these factors significantly increased in the TMP/SMZ receiver group, compared to other groups receiving TMP/SMZ-NLCs, PEG-TMP/SMZ-NLCs, and PBS (Figure 8, Table S8). The higher efficacy of PEG-TMP/SMZ-NLCs, compared to TMP/SMZ-NLCs, in decreasing the side-effects of the drugs could be due to the higher blood circulation stability of PEG-TMP/SMZ-NLCs and release of the drugs for a longer time [66].

The results of toxicity were confirmed by histopathological studies, in which more liver cell necrosis was observed in the TMP/SMZ receiver group (Figure 7B). In addition, the therapeutic effects of the formulations were evaluated in terms of antibacterial effects. The results demonstrated that the numbers of viable bacteria in TMP/SMZ, TMP/SMZ-NLCs, and PEG-TMP/SMZ-NLCs receiver groups were 10^5^, 10^3^, and 10^2^ CFU/mL, respectively. These results indicated the efficacy of PEG-TMP/SMZ-NLCs, compared to TMP/SMZ-NLCs and TMP/SMZ, in increasing the therapeutic effects of TMP/SMZ. This could be due to the positive effects of PEG on the circulation time of the particles and the profile of drug release.

## 4. Conclusions

This study aimed to improve the oral delivery of TMP/SMZ for the treatment of MRSA skin infections using a PEGylated nanoformulation of the drugs. The nanoformulation was successfully synthesized with the size and LE% of 187 ± 9 nm and 9.8%, respectively, that could release the drugs in a controlled manner at both pH 1.2 (64%) and 6.8 (52%). The nanoformulation could significantly decrease the toxicity of the drugs by 2.4-fold and demonstrated high stability with time. In addition, PEG-TMP/SMZ-NLCs could improve the intestinal permeability of the drugs in vitro by 54%. The nanoformulation was found efficient in increasing the antibacterial effects of the drugs against the MRSA bacterium by eightfold in vitro. The efficacy results of PEG-TMP/SMZ-NLCs in improving the oral delivery of the drugs in the treatment of MRSA skin infection in vivo demonstrated that PEG-TMP/SMZ-NLCs could decrease the toxicity of the drugs by ~5-fold, which was confirmed by histopathological studies. In addition, PEG-TMP/SMZ-NLCs could enhance the antibacterial effects of the drugs after oral administration in the infected mice by three orders of magnitude. According to these results, it can be concluded that PEG-TMP/SMZ-NLCs could be considered a promising carrier for the oral delivery of TMP/SMZ for the treatment of MRSA skin infection. Oral drug delivery is an ideal route to achieve therapeutic and prophylactic effects against diseases. For this purpose, NLCs have demonstrated great promise as these nanoparticles can protect entrapped drugs from degrading enzymes and harsh pH conditions, adhere to the intestinal mucus, and inhibit the P-gp-mediated efflux. In addition, the surface of NLCs can be modified with polymers and peptides to further improve their oral bioavailability. Moreover, these nanoparticles release the entrapped drugs in a controlled manner, resulting in a decrease in the side-effects of the loaded drugs. Therefore, NLCs have the commercialization potential for the preparation of oral formulations.

## Figures and Tables

**Table 1:** Components of the lipid and aqueous phases used for the preparation of nanostructured lipid carriers (NLCs) and polyethylene glycol (PEG)ylated NLCs.

Ingredients	NonPEGylated NLCs (% *w*/*v*)	PEGylated NLC (% *w*/*v*)
**Lipid phase**		
Lecithin	2	2
Monostearin	1	1
Soybean oil	0.4	0.4
TMP/SMZ	0.5	0.5
DSPE-PEG2000	0	0.6
**Aqueous phase**		
Tween 80	0.1	0.1
Poloxamer 188	0.2	0.2
Water	95.8	95.2

**Table 2:** Size, polydispersity index (PDI), zeta potential, EE%, and LE% of various nanoparticles obtained by Zetasizer and ultra-high performance liquid chromatography (UHPLC).

Formuation	Size (nm)	PDI	Zeta Potential (mV)	EE%	LE%
NLC	184 ± 7	0.332 ± 0.014	−25 ± 1.4	N/A	N/A
PEG-NLC	170 ± 10	0.314 ± 0.013	−20 ± 1.3	N/A	N/A
TMP/SMZ-NLC	198 ± 11	0.273 ± 0.011	−19 ± 1	86.2	10.4
PEG-TMP/SMZ-NLC	187 ± 9	0.244 ± 0.01	−13 ± 0.8	93.3	9.8

**Table 3:** Drug release, cell toxicity, and LE% for TMP/SMZ-NLCs and PEG-TMP/SMZ-NLCs at the preparation time and 3 months after preparation.

	Drug Release (%)				Cell Viability (IC_50_)		LE%	
	P.T		T.M.P.T		P.T	T.M.P.T	P.T	T.M.P.T
	pH		pH					
	1.2	6.8	1.2	6.8				
TMP/SMZ-NLCs	86%	73%	91%	76.3%	25.4	27.8	10.4	9.7
PEG-TMP/SMZ-NLCs	64%	52%	67%	54.5%	40.9	43.1	9.8	9.1

P.T: Preparation time; T.M.P.T: Three months after the preparation time.

**Table 4:** Size and polydispersity index (PDI) for TMP/SMZ-NLCs and PEG-TMP/SMZ-NLCs on the production time (P.T) and three months after the preparation (T.M.P.T).

	Size (nm)		Polydispersity Index (PDI)	
	P.T	T.M.P.T	P.T	T.M.P.T
TMP/SMZ-NLCs	198 ± 11	212 ± 12	0.273 ± 0.011	0.311 ± 0.013
PEG-TMP/SMZ-NLCs	187 ± 9	195 ± 11	0.244 ± 0.01	0.267 ± 0.010

P.T: Preparation time; T.M.P.T: Three months after the preparation time.

## Data Availability

The raw data will be available on request.

## Supplementary Materials

The following supporting information can be downloaded at: https://www.mdpi.com/article/10.3390/pharmaceutics14081668/s1, Figure S1: The Higuchi model of TMP and SMZ release from (A) TMP/SMZ-NLCs (pH 1.2), (B) TMP/SMZ-NLCs (pH 6.8), (C) PEG-TMP/SMZ-NLCs (pH 1.2), and (D) PEG-TMP/SMZ-NLCs (pH 6.8); Figure S2: The stability of TMP/SMZ-NLCs and PEG-TMP/SMZ-NLCs in fetal bovine serum (FBS) over 5 h at 37 °C. Data is expressed as mean ± SD (n = 3); Table S1: Formulation and optimization of non-PEGylated nanostructured lipid carriers (NLCs) using various monostearin/lecithin ratios; Table S2: Formulation and optimization of PEGylated NLC nanoparticles using various PEG/(lecithin + monostearin) lipid ratios; Table S3: Formulation and optimization of drug-loaded PEGylated and non-PEGylated NLC nanoparticles using various drug (TMP/SMZ) concentrations.; Table S4: Values of cumulative % drug released, % drug remaining, square root time, log cumulative % drug remaining, log time, log cumulative % drug released, % drug released, cube root of % drug remaining (Wt) and W0-Wt parameters used to determine the kinetics of the drug release from TMP/SMZ-NLCs at pH 1.2. W0 and Wt are the initial and remaining amount of drug in the pharmaceutical dosage form at times 0 and t, respectively; Table S5: Values of cumulative % drug released, % drug remaining, square root time, log cumulative % drug remaining, log time, log cumulative % drug released, % drug released, cube root of % drug remaining (Wt) and W0-Wt parameters used to determine the kinetics of the drug release from TMP/SMZ-NLCs at pH 6.8. W0 and Wt are the initial and remaining amount of drug in the pharmaceutical dosage form at times 0 and t, respectively; Table S6: Values of cumulative % drug released, % drug remaining, square root time, log cumulative % drug remaining, log time, log cumulative % drug released, % drug released, cube root of % drug remaining (Wt) and W0-Wt parameters used to determine the kinetics of the drug release from PEG-TMP/SMZ-NLCs at pH 1.2. W0 and Wt are the initial and remaining amount of drug in the pharmaceutical dosage form at times 0 and t, respectively; Table S7: Values of cumulative % drug released, % drug remaining, square root time, log cumulative % drug remaining, log time, log cumulative % drug released, % drug released, cube root of % drug remaining (Wt) and W0-Wt parameters used to determine the kinetics of the drug release from PEG-TMP/SMZ-NLCs at pH 6.8. W0 and Wt are the initial and remaining amount of drug in the pharmaceutical dosage form at times 0 and t, respectively; Table S8: Values of ALT, AST, BUN, and creatinine in control, TMP/SMZ, TMP/SMZ-NLCs, and PEG-TMP/SMZ-NLCs receiver groups. The values are expressed as mean ± SD from three independent experiments.
