# Supplementary material for: A PEGylated Nanostructured Lipid Carrier for Enhanced Oral Delivery of Antibiotics
Source: Pharmaceutics. 2022 Aug 11;14(8):1668. doi: 10.3390/pharmaceutics14081668 (PMC9415149; doi:10.3390/pharmaceutics14081668)
Supplement: Supplementary file 1 [file pharmaceutics-14-01668-s001.zip › pharmaceutics-1854850-supplementary.pdf]

Supplemental Information: **A PEGylated nanostructured lipid carrier for enhanced oral delivery of antibiotics**

Figure S1: The Higuchi model of TMP and SMZ release from **A**) TMP/SMZ-NLCs (pH 1.2), **B**) TMP/SMZ-NLCs (pH 6.8), **C**) PEG-TMP/SMZ-NLCs (pH 1.2), and **D**) PEG-TMP/SMZ-NLCs (pH 6.8).

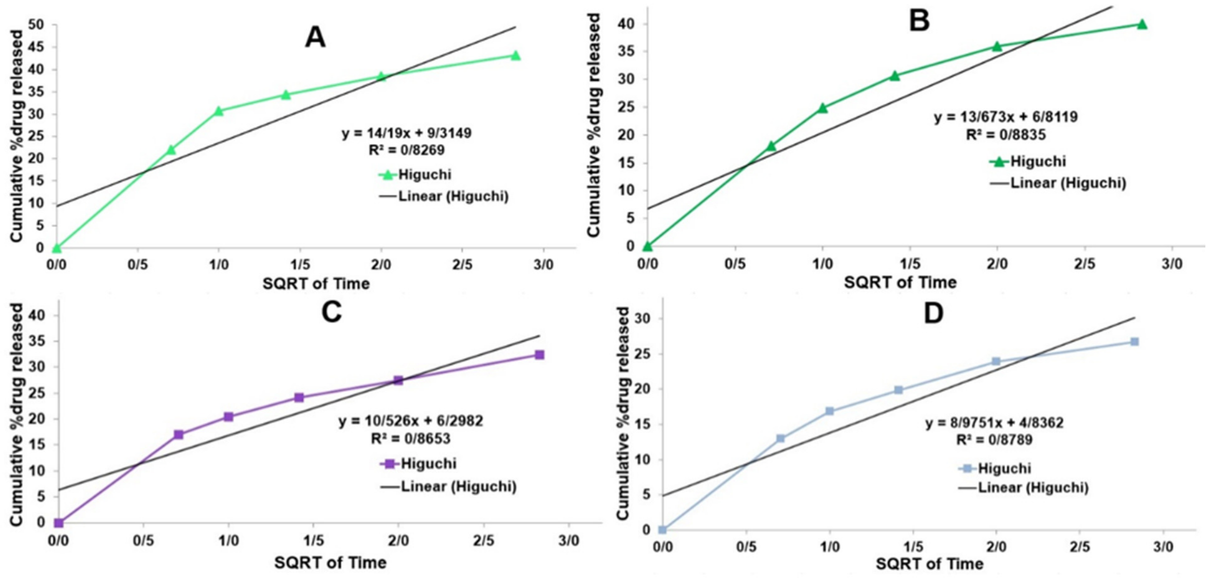

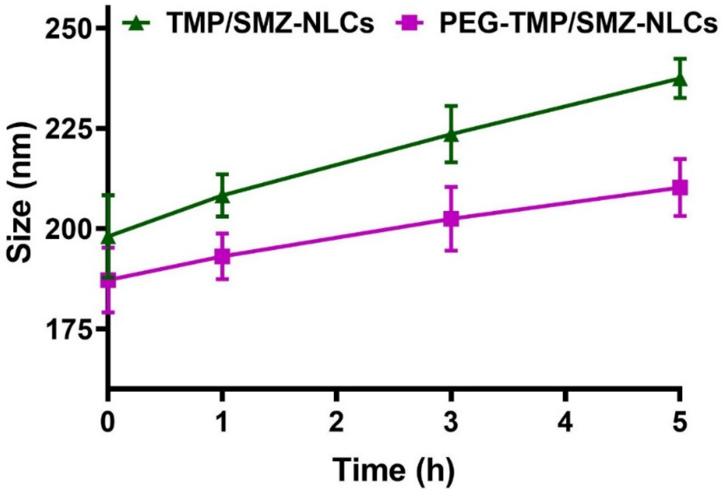

Figure S2: The stability of TMP/SMZ-NLCs and PEG-TMP/SMZ-NLCs in fetal bovine serum (FBS) over 5 h at 37 °C. Data is expressed as mean ± SD (n = 3).

Optimization of the trimethoprim, sulfamethoxazole-loaded polyethylene glycol (PEG)ylated nanostructured lipid carrier (PEG-TMP/SMZ-NLCs) formulation

To optimize the preparation of PEG-TMP/SMZ-NLCs, the formulation composition of nanostructured lipid carrier (NLC) was optimized in terms of size and polydispersity index (PDI) according to the different weight ratios of lecithin and monostearin (Table S1). The optimized formulation was selected based on the minimum size and PDI. The optimized formulation with the minimum size and PDI values (Table S1, N7) was then optimized against 3 different concentrations of DSPE-PEG2000 (Table S2). For this purpose, the 3 concentrations of DSPE-PEG2000 were mixed with N7 separately, and the optimized concentration with minimum size and PDI was selected to mix with 3 different concentrations of the drugs [1] to find the optimized formulation according to their encapsulation efficiency (EE%, Table S3).

Table S1: Formulation and optimization of non-PEGylated nanostructured lipid carriers (NLCs) using various monostearin/lecithin ratios.

Code	Monostearin/Lecithin ratio (w/w%)	Size (nm)	PDI
**N1**	0.3:2	274 ± 14.3	0.44 ± 0.025
**N2**	0.3:1.5	256 ± 14.0	0.34 ± 0.02
**N3**	0.3:1	243 ± 13.1	0.33 ± 0.015
**N4**	0.6:2	254.1 ± 12.3	0.34 ± 0.015
**N5**	0.6:1.5	213.2 ± 10.8	0.36 ± 0.017
**N6**	0.6:1	204.4 ± 10.2	0.41 ± 0.016
**N7**	1:2	184 ± 7	0.332 ± 0.014
**N8**	1:1.5	239 ± 12.3	0.35 ± 0.017
**N9**	1:1	258.6 ± 12.4	0.38 ± 0.01

PDI: Polydispersity index

Table S2: Formulation and optimization of PEGylated NLC nanoparticles using various PEG/(lecithin + monostearin) lipid ratios.

Code	DSPE-PEG2000/lipid ratios (w/w%)	Size (nm)	PDI
**N7P1**	0.2/3	238.4 ± 10.1	0.374 ± 0.017
**N7P2**	0.4/3	214.3 ± 11	0.368 ± 0.014
**N7P3**	0.6/3	170 ± 10	0.314 ± 0.013

Table S3: Formulation and optimization of drug-loaded PEGylated and non-PEGylated NLC nanoparticles using various drug (TMP/SMZ) concentrations.

Code	Drug (w/w%)	Size (nm)	PDI	EE%
**N7C1**	0.125	219.5 ± 10.8	0.346 ± 0.017	76
**N7C2**	0.25	227 ± 11.1	0.234 ± 0.011	79
**N7C3**	0.5	198 ± 11	0.273 ± 0.011	86.2
**N7P3C1**	0.125	213.4 ± 10.1	0.364 ± 0.018	81
**N7P3C2**	0.25	219.0 ± 10.1	0.248 ± 0.030	88
**N7P3C3**	0.5	187 ± 9	0.244 ± 0.01	93.3

EE%: Encapsulation efficiency (%)

Table S4: Values of cumulative % drug released, % drug remaining, square root time, log cumulative % drug remaining, log time, log cumulative % drug released, % drug released, cube root of % drug remaining (W_t) and W_0-W_t parameters used to determine the kinetics of the drug release from TMP/SMZ-NLCs at pH 1.2. W_0 and W_t are the initial and remaining amount of drug in the pharmaceutical dosage form at times 0 and t, respectively.

**Time (h)**	**cumulative % drug released**	**% drug remaining**	**Square root time**	**log Cumu % drug remaining**	**log time**	**log Cumu % drug released**	**% Drug released**	**Cube Root of % drug Remaining (Wt)**	**Wo-Wt**
0	0	100	0.00	2.00	0.00	0.00	100	4.64	0.00
0.5	22	78	0.71	1.89	-0.30	1.34	9.13	4.27	0.37
1	30.6	69.4	1.00	1.84	0.00	1.49	8.67	4.11	0.53
2	34.4	65.6	1.41	1.82	0.30	1.54	3.77	4.03	0.61
4	38.5	61.5	2.00	1.79	0.60	1.59	4.1	3.95	0.70
8	43.2	56.8	2.83	1.75	0.90	1.64	4.7	3.84	0.80

Table S5: Values of cumulative % drug released, % drug remaining, square root time, log cumulative % drug remaining, log time, log cumulative % drug released, % drug released, cube root of % drug remaining (W_t) and W_0-W_t parameters used to determine the kinetics of the drug release from TMP/SMZ-NLCs at pH 6.8. W_0 and W_t are the initial and remaining amount of drug in the pharmaceutical dosage form at times 0 and t, respectively.

**Time (h)**	**cumulative % drug released**	**% drug remaining**	**Square root time**	**log Cumu % drug remaining**	**log time**	**log Cumu % drug released**	**% Drug released**	**Cube Root of % drug Remaining (Wt)**	**Wo-Wt**
0	0	100	0.00	2.00	0.00	0.00	100	4.64	0.000
0.5	18	82	0.71	1.91	-0.30	1.26	-	4.34	0.30
1	24.9	75.1	1.00	1.88	0.00	1.40	6.93	4.22	0.42
2	30.7	69.3	1.41	1.84	0.30	1.49	5.77	4.11	0.53
4	35.9	64.1	2.00	1.81	0.60	1.56	5.23	4.00	0.64
8	40	60	2.83	1.78	0.90	1.60	4.07	3.92	0.73

Table S6: Values of cumulative % drug released, % drug remaining, square root time, log cumulative % drug remaining, log time, log cumulative % drug released, % drug released, cube root of % drug remaining (W_t) and W_0-W_t parameters used to determine the kinetics of the drug release from PEG-TMP/SMZ-NLCs at pH 1.2. W_0 and W_t are the initial and remaining amount of drug in the pharmaceutical dosage form at times 0 and t, respectively.

**Time (h)**	**cumulative % drug released**	**% drug remaining**	**Square root time**	**log Cumu % drug remaining**	**log time**	**log Cumu % drug released**	**% Drug released**	**Cube Root of % drug Remaining (Wt)**	**Wo-Wt**
0	0	100	0.00	2.00	0.00	0.000	100	4.64	0.00
0.5	17	83	0.71	1.92	-0.30	1.230	-	4.36	0.28
1	20.4	79.6	1.00	1.90	0.00	1.310	3.4	4.30	0.34
2	24.2	75.8	1.41	1.88	0.30	1.384	3.8	4.23	0.41
4	27.5	72.5	2.00	1.86	0.60	1.439	3.27	4.17	0.47
8	32.4	67.6	2.83	1.83	0.90	1.511	4.93	4.07	0.57

Table S7: Values of cumulative % drug released, % drug remaining, square root time, log cumulative % drug remaining, log time, log cumulative % drug released, % drug released, cube root of % drug remaining (W_t) and W_0-W_t parameters used to determine the kinetics of the drug release from PEG-TMP/SMZ-NLCs at pH 6.8. W_0 and W_t are the initial and remaining amount of drug in the pharmaceutical dosage form at times 0 and t, respectively.

Time (h)	cumulative % drug released	% drug remaining	Square root time	log Cumu % drug remaining	log time	log Cumu % drug released	% Drug released	Cube Root of % drug Remaining (Wt)	Wo-Wt
0	0	100	0.00	2.00	0.00	0.00	100	4.64	0.00
0.5	13	87	0.71	1.94	-0.30	1.11	-	4.43	0.21
1	16.9	83.1	1.00	1.92	0.00	1.23	3.87	4.36	0.28
2	19.8	80.2	1.41	1.90	0.30	1.30	2.97	4.31	0.33
4	24	76	2.00	1.88	0.60	1.38	4.13	4.24	0.41
8	26.7	73.3	2.83	1.87	0.90	1.43	2.73	4.19	0.46

Table S8: Values of ALT, AST, BUN, and creatinine in control, TMP/SMZ, TMP/SMZ-NLCs, and PEG-TMP/SMZ-NLCs receiver groups. The values are expressed as mean ± SD from three independent experiments.

Control	TMP/SMZ	TMP/SMZ-NLCs	PEG-TMP/SMZ-NLCs
**ALT**			
15.8 ± 1.2	88.5 ± 5	21.4 ± 2.7	18 ± 1
**AST**			
26.6 ± 3.1	121.9 ± 5	37.1 ± 3.8	30.4 ± 5.4
**BUN**			
24.8 ± 2.2	171.8 ± 7.1	27.2 ± 6	23.1 ± 2.1
**Creatinine**			
0.5 ± 0.04	2.4 ± 0.1	0.64 ± 0.1	0.61 ± 0.045
